# Effects of chronic sleep deprivation on the bone mineral density, bone microarchitecture and body weight of mice: exploratory study

**DOI:** 10.3389/fphys.2026.1795836

**Published:** 2026-05-05

**Authors:** Weiwei Xiang, Zhen Lv, Xinwen Min, Junhao Zhang, Handong Yang, Jun Chen, Jishun Chen, Dongfeng Li, Hao Xu, Chao Luo, Wenwen Wu

**Affiliations:** 1 School of Public Health, Hubei University of Medicine, Shiyan, Hubei, China; 2 Sinopharm Dongfeng General Hospital (Hubei Clinical Research Center of Hypertension), Hubei University of Medicine, Shiyan, Hubei, China; 3 Department of Public Health, Quanzhou Matemal and Child Health Hospital (Quanzhou Children’s Hospital), Quanzhou, Fujian, China; 4 School of Basic Medical Sciences, Hubei University of Medicine, Shiyan, Hubei, China; 5 Center of Health Administration and Development Studies, Hubei University of Medicine, Shiyan, Hubei, China

**Keywords:** bone microarchitecture, bone mineral density, chronic sleep deprivation, exploratory study, mice

## Abstract

**Objective:**

This study aimed to investigate the long-term effects of chronic sleep deprivation (CSD) on the bone mineral density (BMD), bone microarchitecture, biomechanical properties and body weight of middle-aged and older male C57BL/6 mice.

**Methods:**

Twenty-one 8-month-old mice were randomly assigned to a CSD group (n = 9) or a control group (n = 12). The CSD group was subjected to sleep deprivation using an automated sleep deprivation apparatus for 3 months. Trabecular bone microarchitecture at the distal femur was evaluated by microcomputed tomography. Femoral biomechanical properties were assessed using a three-point bending test, and body weight was monitored weekly throughout the experiment.

**Results:**

Three months of CSD significantly reduced BMD, bone volume (BV) and trabecular thickness (Tb.Th) compared with those of the control group (all *P* < 0.05). Trabecular separation (Tb.Sp) and bone surface (BS) were also significantly decreased (*P* < 0.05). No significant differences were observed in trabecular number (Tb.N), bone volume fraction (BV/TV) or other microarchitectural parameters. Three-point bending test showed no statistically significant differences in any biomechanical indices, including maximum load, bending stiffness and elastic modulus (all *P* > 0.05), between the two groups. Body weight monitoring revealed that the CSD group had significantly lower body weight at the early stage of the experiment (week 1) and at week 8 compared with the controls (*P* < 0.05). No significant differences were observed at most other time points, showing a fluctuating pattern with baseline differences.

**Conclusion:**

Three months of CSD led to decreased BMD and trabecular thinning in middle-aged and older male mice. No significant deterioration of overall bone biomechanical properties was observed, suggesting the presence of compensatory mechanisms in bone. The effect of sleep deprivation on body weight was intermittent rather than sustained. These findings provide experimental evidence for a comprehensive understanding of the relationship between sleep duration and skeletal health.

## 1 Introduction

Osteoporosis (OP) is a systemic skeletal disease characterised by reduced bone mass and deterioration of bone microarchitecture, leading to increased bone fragility and an elevated risk of fractures (Zhang et al., 2025). Amongst individuals aged 50 years and older, the lifetime risk of osteoporotic fractures is estimated to be 40%–50% in women and 13%–22% in men (Johnell and Kanis, 2005). The incidence of OP and related fractures increases exponentially after the age of 50 years (Hsieh et al., 2025), and declines in bone mass and bone strength are closely associated with advancing age (Otsuka et al., 2025; Wang et al., 2024). OP poses a major threat to global health. More than 8.9 million osteoporotic fractures occur worldwide each year, corresponding to one fracture approximately every 3 seconds (Johnston and Dagar, 2020). With the ongoing aging of the global population, the demand for OP prevention and management continues to rise and the associated individual and socioeconomic burdens are increasing, placing substantial challenges on public health systems.

Numerous studies investigated the factors associated with OP. Although a wide range of influencing factors have been identified, many of them remain at an exploratory stage. Sleep is one of the potential factors associated with OP development. It is closely related to daily life and represents a vital physiological process that influences metabolic regulation, energy balance and dietary behaviour (Hall, 2026). Adequate sleep is therefore essential for human health. Long-term sleep insufficiency has been shown to increase mortality and morbidity, reduce quality of life and impair family well-being. Owing to factors such as increased intake of energy-dense foods, extensive use of the internet and media, high-intensity lifestyles, great exposure to environmental light and prolonged physical inactivity (Morrison et al., 2022), modern populations are increasingly experiencing chronic sleep deprivation (CSD), which contributes to deteriorating sleep quality. CSD generally refers to the repeated occurrence of partial sleep deprivation (SD) over an extended period.

Sleep duration is associated with OP. Duan et al (Duan et al., 2022) established an SD rat model and found that SD reduced bone mineral density (BMD), leading to decreased bone mass and bone microarchitecture deterioration, thereby increasing the risk of OP. An interventional study investigated the effects of severe SD (2 hours of sleep per night for three consecutive nights) on bone turnover markers in 10 healthy male soldiers. The study showed that bone formation, represented by bone-specific alkaline phosphatase, decreased after one night of SD, whereas bone resorption, represented by the C-terminal telopeptide of type I collagen (CTX) and tartrate-resistant acid phosphatase, increased after two nights of SD. This uncoupling of bone turnover, where bone resorption exceeds formation, is expected to reduce BMD if sustained over time (Staab et al., 2019).

Contradictory findings have also been reported. Yamaura et al (Yamaura et al., 2023) found no significant association between SD and BMD in a clinical survey. Another study reported that insufficient sleep was not significantly correlated with reduced BMD in older adults (Swanson et al., 2021). Using a sleep duration of 8–9 hours per day as a reference, a large-scale clinical survey in China found that short and long sleep durations were associated with an increased risk of developing OP (Wang et al., 2023).

Although some studies explored the relationship between sleep and OP, no consistent conclusions have been reached, and the potential mechanisms linking bone metabolism to CSD remain unclear and warrant further investigation. Compared with clinical studies, animal experiments offer better control over confounding factors and are therefore a preferred approach for investigating the effects of sleep on OP. Experimental animals commonly used to establish OP models include mice, rats, pigs, sheep and nonhuman primates (Maenz et al., 2020; Saito et al., 2015; Seo et al., 2023; Xiao et al., 2020; Zhang et al., 2024). Among them, large animals such as pigs and monkeys are limited in use because of high housing costs, management difficulties and low reproductive efficiency. By contrast, mice, with their small body size, low cost and ease of maintenance, have become the preferred animal model for OP research (Liu et al., 2024).

Previous epidemiological studies on the relationship between sleep duration and osteoporosis primarily focused on general populations or postmenopausal women; only few specifically targeted middle-aged and older men. Therefore, the present work used the algorithm described in reference (Dutta and Sengupta, 2016) to convert the lifespan of C57BL/6 mice to human-equivalent ages. According to this conversion, C57BL/6 mice aged 32, 36 and 40 weeks correspond to human ages of 62.4, 70.2 and 78 years, respectively. On this basis, we established an SD model using 8-month-old male C57BL/6 mice. By comparing BMD, radiographic and histological microarchitecture and biomechanical bone strength between the CSD group and normal activity control group, the present study aimed to explore the effects of sleep duration on bone mass, bone strength and bone metabolism in mice.

## 2 Methods

### 2.1 Experimental animals and housing conditions

Male C57BL/6 mice (8 months old) were provided by the Animal Experiment Center of Hubei University of Medicine. All animals were housed at the Animal Experiment Center of Hubei University of Medicine. The mice in the control group were maintained under controlled temperature (22 °C–24 °C) and humidity (50%–60%) with a 12-hour light–dark cycle. The mice in the CSD group were housed under natural light conditions, with lighting following the natural day–night rhythm (lights on at dawn and off at dusk) and the ambient temperature maintained at 22 °C. All mice were fed standard chow and had free access to food and water (including during the CSD intervention). Food and water were provided ad libitum except for a 24-hour fasting period prior to blood sample collection.

### 2.2 Experimental grouping and procedures

Twenty-one 8-month-old male C57BL/6 mice were randomly assigned into the CSD group (n = 9) or control group (n = 12). Body weight was considered during group allocation to minimise baseline differences; however, a residual imbalance remained, likely due to the small sample size. Each mouse was individually marked with an ear tag. After one week of acclimatisation, the mice in the CSD group were placed in the CSD experimental environment for 1 week of adaptive training, whilst the control mice were housed in the control environment for the same period. Following adaptive training, all mice were weighed, and body weight was subsequently recorded on a weekly basis. At 1, 2 and 3 months, seven mice were euthanised at each time point (control group: 4; CSD group: 3), and bilateral femora were collected for microcomputed tomography (micro-CT) scanning and biomechanical testing.

### 2.3 Establishment of the chronic sleep deprivation model

CSD was induced using a small/large rodent SD apparatus (Model XR-XS108-I, Shanghai Xinruan Information Technology Co., Ltd.). The mice in the SD cages were able to move freely. When the mice became drowsy and attempted to sleep, a rotating bar inside the cage rotated at 10 RPM to gently disturb them. All parameters, including rotation direction and speed, were set via the touchscreen controller, allowing adjustment to reduce environmental adaptation by the animals. The rotating bar provided 360°disturbance with no blind spots, enabling SD without direct human intervention and thereby establishing the CSD model. The mice subjected to CSD were placed in the SD environment daily at 16:00 and returned to standard cages at 10:00 the following day. The XR-XS108-I apparatus has been widely used in published sleep deprivation studies (Hua et al., 2022; Li et al., 2024).Similar to previous reports, the rotating bar was set at 10 RPM with random direction changes to prevent habituation. Periodic behavioural observations conducted by the experimenters confirmed that mice in the CSD group remained awake and active throughout the entire intervention period.

### 2.4 Micro-CT scanning

At 1, 2 and 3 months after the SD intervention, mice were anaesthetised with pentobarbital sodium, and bilateral femora were rapidly dissected and carefully cleaned of surrounding soft tissues. The specimens were then positioned on the sample holder with the long axis aligned consistently for all samples. Micro-CT scanning was performed using a Quantum GX2 system (PerkinElmer, Japan). The scanning protocol was set as follows: voltage 50 kVp, current 100 μA and effective pixel size 36 μm. All specimen were scanned under identical conditions to ensure comparability. Each femur was scanned once. Three-dimensional reconstruction and quantitative analysis were carried out using the built-in analysis software of the manufacturer. Trabecular bone parameters, including BMD, bone volume, and trabecular thickness, were automatically calculated based on the full three-dimensional (3D) dataset. The left and right femurs of each mouse were analysed separately, and the mean of the two values was used as the final value for that animal. All quantitative parameters were derived from the complete 3D volume rather than from selected two-dimensional slices, thereby minimising sampling bias and ensuring a comprehensive evaluation of bone microarchitecture.

### 2.5 Three-point bending test

Femoral biomechanical properties were assessed using an Instron E10000 universal testing machine. During testing, each femur was placed on two supporting bars, with the loading rod positioned at the midpoint and a span of 6 mm. All specimens were loaded at a constant rate of 3 mm/min until fracture. The maximum bending force and maximum bending deformation prior to fracture were recorded, and bending stiffness, elastic modulus and bending stress parameters were calculated.

### 2.6 Animal welfare, monitoring and ethics

The study protocol was approved by the Animal Ethics Committee of Hubei University of Medicine (Approval No. 2023-RE-015). Efforts to minimise suffering included gentle handling, environmental enrichment (nesting material), and daily health checks. Predefined humane endpoints (e.g., >20% weight loss, severe lethargy) were not triggered. No serious adverse events occurred.

All mice were euthanised between 9:00 AM and 10:00 AM. At each terminal time point, mice were first deeply anaesthetised via intraperitoneal injection of sodium pentobarbital. Depth of anaesthesia was confirmed by the absence of pedal withdrawal and corneal reflexes. While the animals were maintained under this surgical plane of anaesthesia, euthanasia was completed by exsanguination via orbital blood collection.

### 2.7 Statistical analysis

All data were analysed using SPSS version 26.0. Quantitative data conforming to a normal distribution were expressed as mean ± standard deviation (`c ± SD), and comparisons between two independent groups were performed using the t-test. For non-normally distributed quantitative data, the Mann–Whitney U test (two independent samples) was applied as the nonparametric test. A two-sided *P* value < 0.05 was considered statistically significant.

## 3 Results

### 3.1 Effects of chronic sleep deprivation on bone microarchitecture and bone mass in mice

Comparison of bone microarchitecture and bone mass parameters between the CSD and control groups at 3 months revealed the significant negative effects of SD on skeletal health. BMD was significantly lower in the CSD group (2194.84 ± 134.48 mg/cm³) than in the control group (2387.48 ± 115.22 mg/cm³, *P* = 0.002). Bone volume (BV; 0.91 ± 0.20 mm³ vs. 1.12 ± 0.19 mm³, *P* = 0.022) and Tb.Th (0.07 ± 0.01 mm vs. 0.08 ± 0.01 mm, *P* = 0.004) were also markedly reduced. Tb.Sp (0.24 ± 0.04 mm vs. 0.27 ± 0.03 mm, *P* = 0.037) and bone surface area (BS; 7.33 ± 3.60 mm² vs. 10.75 ± 3.74 mm², *P* = 0.049) were significantly decreased. However, no significant differences in Tb.N, BS/BV or BV/TV were observed between the two groups. This finding indicates that CSD primarily impaired bone density, bone volume and trabecular thickness without broadly affecting trabecular number or overall bone architecture. Detailed results are shown in **Table 1**.

**Table 1:** Comparison of bone parameters between chronic sleep deprivation (CSD) and control mice at 3 months.

Parameter	CSD group	Control group	*t*-value	*P*-value
BMD(mg/cm^3^)	2194.84 ± 134.48	2387.48 ± 115.22	3.532	0.002
Tb.Th(mm)	0.07 ± 0.01	0.08 ± 0.01	3.233	0.004
Tb.Sp(mm)	0.24 ± 0.04	0.27 ± 0.03	2.244	0.037
Tb.N(1/mm)	0.89 ± 0.36	1.13 ± 0.42	1.332	0.199
BS/BV(1/mm)	48.85 ± 4.39	51.83 ± 8.19	0.989	0.335
BV/TV(%)	6.97 ± 3.63	10.10 ± 4.55	1.696	0.106
BS(mm^2^)	7.33 ± 3.60	10.75 ± 3.74	2.108	0.049
BV(mm^3^)	0.91 ± 0.20	1.12 ± 0.19	2.493	0.022

BMD, bone mineral density; BV/TV, bone volume fraction; BS/BV, bone surface area over bone volume; Tb.Th, trabecular thickness; Tb.N, trabecular number; Tb.Sp, trabecular separations.

### 3.2 Micro-CT-based morphological comparison of femoral trabecular bone between the experimental and control groups

**Figure 1:**
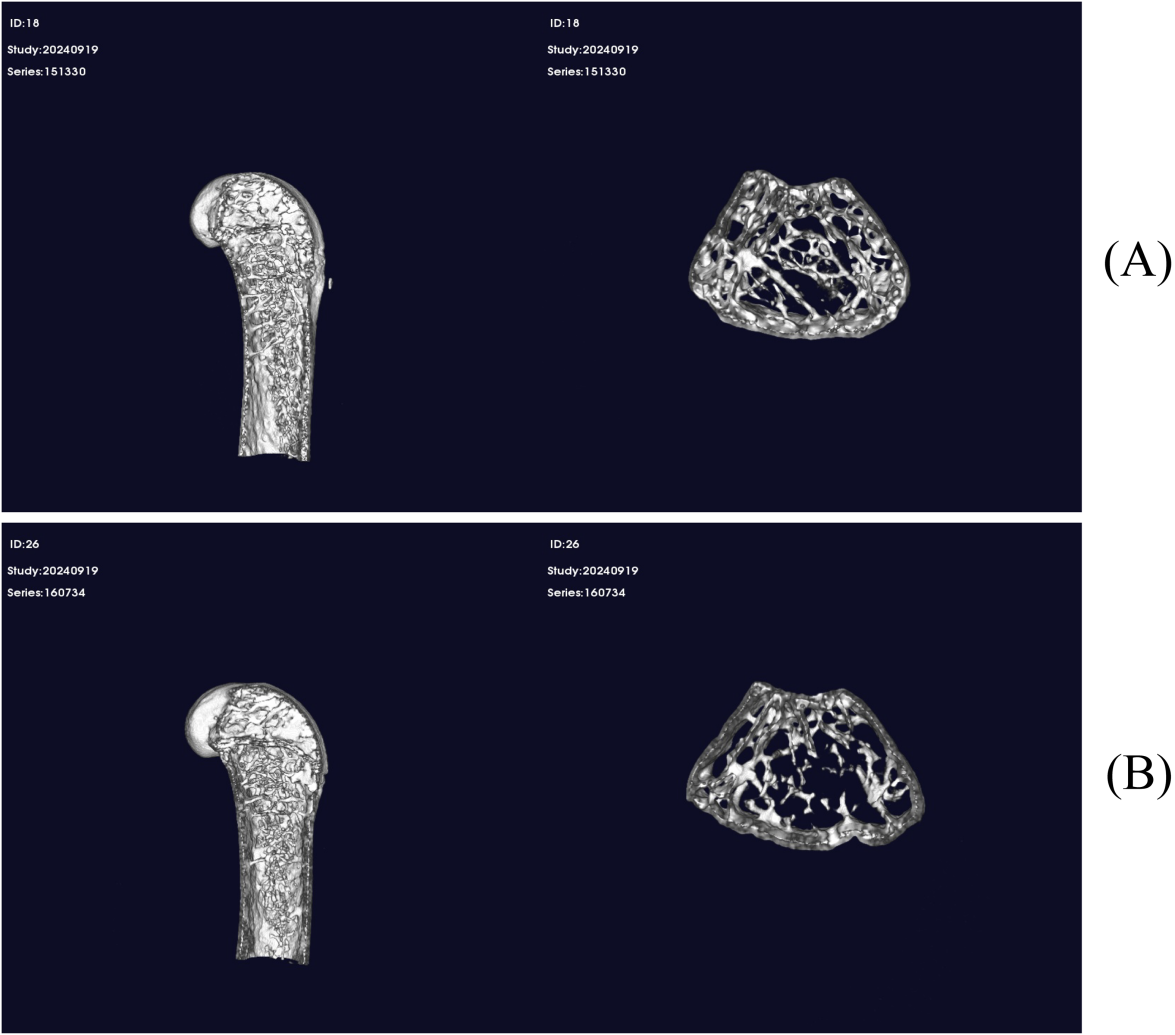
Micro-CT 3D reconstruction images showing the effects of chronic sleep deprivation on femoral microarchitecture in mice. **(A)**, Control group; **(B)**, CSD group.

In this work, 3D reconstruction and coronal plane images of the distal femoral trabecular region were obtained by micro-CT to provide direct morphological evidence of bone microstructural alterations (**Figure 1**). Compared with those in the control group, the mice in the CSD group exhibited pronounced deterioration of femoral bone microarchitecture.

- Control group: In the control group, the trabecular bone in the distal femoral metaphysis exhibited a dense, continuous and well-organised trabecular network. Trabeculae were thick and well connected, forming an intact plate-like porous structure with clearly defined supporting architecture. The cortical bone appeared uniform in thickness and continuous.
- CSD group: The distal femoral trabecular region in the CSD group showed marked bone loss and structural deterioration. The trabecular network was evidently sparse and thin with disrupted continuity, characterised by multiple trabecular fractures and isolated free ends. The original plate-like structure was disrupted, resulting in a fragile, rod-like network. Cortical bone appeared visually thinner compared with that in the control group.

These morphological observations were in agreement with the quantitative micro-CT findings, supporting the observed structural alterations. The trabecular thinning, disruption and reduction observed in the CSD group directly corresponded to the significant decrease in Tb.Th and BV. Moreover, the overall sparse appearance of the trabecular network was in agreement with the significantly reduced BMD. Collectively, these findings confirm that 3 months of CSD resulted in distinct and observable damage to bone microarchitecture.

### 3.3 Effects of chronic sleep deprivation on bone biomechanical properties

Femoral biomechanical properties were evaluated using three-point bending test. Despite the deterioration observed in bone microstructural parameters, 3 months of CSD did not lead to statistically significant changes in the overall mechanical properties of the femur. Compared with the control group, the CSD group exhibited slightly lower values of maximum load and bending stiffness (e.g. stiffness: 53.59 ± 16.44 N/mm vs. 59.74 ± 18.32 N/mm), whereas the elastic modulus and bending stress were slightly higher. However, no statistically significant differences were observed between the two groups for any biomechanical parameter, with P values ranging from 0.155 to 0.803. These findings suggest that within the duration of this study, the bones may have maintained overall mechanical integrity through compensation in material properties or geometric structure, and the structural changes induced by SD are not yet translated into a measurable decline in biomechanical function. Detailed results are presented in **Table 2**.

**Table 2:** Summary of femoral three-point bending test results in mice from the chronic sleep deprivation and control groups at 3 months.

Parameter	Control group	CSD group	*t*/*Z* value	*P* value
Maximum Load (N)	19.07[16.00,20.90]	15.69[15.34,16.76]	-1.421	0.155
Maximum Deformation (mm)	0.40[0.35,0.57]	0.49[0.34,0.62]	-0.249	0.803
Bending Stiffness (N/mm)	59.74 ± 18.32	53.59 ± 16.44	0.795	0.437
Elastic Modulus (MPa)	803.39[700.75,1376.56]	1029.22[895.37,1465.825]	-1.066	0.286
Bending Stress (MPa)	65.92[55.30,102.69]	81.51[67.68,88.22]	-0.924	0.356

### 3.4 Dynamic changes in body weight during chronic sleep deprivation

The body weight of mice in both groups was tracked over 13 weeks. In the first week, the body weight of the CSD group (32.60 ± 1.52 g) was significantly lower than that of the control group (36.67 ± 1.72 g, *P* < 0.001), and a significantly reduction was again observed at week 8 (*P* = 0.013). In the remaining weeks (weeks 2–7 and 9–13), no significant differences were observed between the two groups, although the mean values in the control group remained slightly higher. These results indicate that CSD transiently exacerbated body weight loss at specific time points but did not induce a persistent or progressive decline, suggesting that SD may intermittently, rather than cumulatively, negatively affect energy metabolism or feeding behaviour. Detailed data are presented in **Table 3**. A significant baseline difference in body weight between the two groups was observed at week 1, which should be considered when interpreting longitudinal weight changes.

**Table 3:** Weekly body weight monitoring of mice in the chronic sleep deprivation and control groups.

Time (weeks)	Body weight in CSD group (g)	Body weight in control group (g)	t value	*P* value
1	32.60 ± 1.52	36.67 ± 1.72	5.615	<0.001
2	34.47 ± 2.22	35.79 ± 2.55	1.242	0.229
3	34.92 ± 2.36	36.65 ± 2.29	1.690	0.107
4	36.01 ± 2.17	36.83 ± 1.59	1.006	0.327
5	36.72 ± 2.08	37.31 ± 1.83	0.569	0.580
6	37.20 ± 2.10	37.56 ± 1.84	0.344	0.737
7	37.23 ± 1.89	38.63 ± 1.97	1.331	0.208
8	35.42 ± 2.26	38.49 ± 1.70	2.911	0.013
9	38.10 ± 2.18	39.18 ± 2.34	0.618	0.563
10	38.03 ± 2.14	39.05 ± 2.51	0.562	0.598
11	37.00 ± 2.25	39.45 ± 2.92	1.199	0.284
12	36.70 ± 2.07	39.70 ± 2.86	1.526	0.187
13	37.53 ± 3.40	40.08 ± 2.08	1.239	0.270

## 4 Discussion

In this study, a CSD model was established using middle-aged male C57BL/6 mice to systematically evaluate the effects of 3 months of SD on skeletal health from multiple perspectives, including bone mass, bone microarchitecture, biomechanical properties and dynamic changes in body weight. Results demonstrated that CSD led to clear microstructural impairments, including reduced BMD, decreased bone volume and trabecular thinning. Meanwhile, overall femoral biomechanical properties remained largely unchanged during the same period. The effects of SD on body weight were intermittent and fluctuating rather than sustained, indicating that CSD may induce temporal, rather than continuous, alterations in energy balance or metabolic regulation.

Firstly, the 3D reconstruction from micro-CT provided robust visual evidence to support the quantitative data. This study revealed significant decreases in BMD, Tb.Th and other parameters in the CSD group and directly observed classic osteoporotic pathological changes in the distal femoral trabecular region, including sparse trabecular networks, fractures and disrupted continuity. These qualitative morphological deteriorations were highly consistent with the declines in quantitative bone parameters, mutually reinforcing each other and collectively confirming that CSD causes substantial damage to bone microarchitecture. This finding strengthens the reliability of the conclusion that insufficient sleep is a risk factor for skeletal health and is consistent with the trabecular deterioration observed by Duan et al (Duan et al., 2022) in animal models. Different from the rapid bone turnover pattern characterised by ‘bone resorption exceeding bone formation’ observed in short-term severe SD studies in humans by Swanson et al (Swanson et al., 2019), the present study revealed more chronic structural changes at the 3-month time point, with no alterations in Tb.N or other parameters. This result suggests that long-term CSD may affect bone metabolism through mechanisms distinct from acute deprivation, and the skeleton may exhibit different adaptive responses to prolonged sleep loss.

Secondly, the three-point bending test did not detect a corresponding decline in bone biomechanical properties, despite the significant deterioration in bone microarchitecture. This ‘structure–function dissociation’ may suggest the presence of compensatory mechanisms. Possible explanations include: (1) remodelling of bone geometry, such as cortical thickness or diameter, may compensate for trabecular bone loss; although morphological observations indicated that the cortical bone in the CSD group appeared visually thinner than before the experiment, the potential changes in material properties require further investigation; and (2) the 3-month intervention period may not have been sufficient to reach the threshold for the overt compromise of macroscopic mechanical performance. These findings imply that when evaluating skeletal risk associated with SD, solely relying on microstructural imaging (including morphology and quantitative parameters) may underestimate early damage; the delayed changes in biomechanical properties suggest the existence of a potential ‘window of intervention’.

Regarding body weight, this study found that its changes were fluctuating, with significant decreases observed at specific time points such as week 8. This finding reflects the complex effects of SD on energy balance, which may be related to the dysregulation of appetite-regulating hormones (e.g. leptin and growth hormone–releasing peptide), alterations in resting energy expenditure or stress responses (e.g. elevated cortisol levels) (Chaput et al., 2023; Feeney et al., 2025). The non-persistent pattern of body weight change suggests that the effects of SD are not linearly cumulative, and the organism may attempt to restore homeostasis during intermittent periods. The progressive deterioration of bone microarchitecture did not fully coincide with the fluctuating body weight pattern, further indicating that SD may impair skeletal health through direct effects on bone metabolism (such as endocrine dysregulation or inflammatory activation), rather than solely via body weight as a mediating factor. Our body weight findings differ from previous studies reporting weight gain associated with sleep deprivation. This discrepancy may be explained by several factors. First, differences in animal age may play an important role. Du et al (Du et al., 2024) used 3-week-old Kunming mice under a similar sleep deprivation protocol (18 h/day deprivation with 6 h opportunity sleep) and observed significant weight gain from week 2 to week 4. In contrast, the present study used 8-month-old C57BL/6 mice and observed only a transient weight decrease at week 8, followed by recovery. These findings suggest that age-related differences in growth and metabolic regulation may influence the physiological response to chronic sleep deprivation, with younger animals more prone to energy storage and older animals exhibiting greater metabolic compensation. Second, variations in sleep deprivation paradigms may also contribute to the discrepant findings. Wang et al (Wang et al., 2014) used a sleep fragmentation model involving frequent tactile stimulation, which frequently activates the sympathetic nervous system and promotes appetite without substantially reducing total sleep duration, thereby promoting sustained weight gain. In contrast, the present study employed a chronic partial sleep deprivation model with a daily 6 h opportunity for sleep which may allow partial metabolic recovery and attenuate cumulative effects on body weight. Finally, the balance between energy expenditure and energy intake may further contribute to the observed differences. Sleep deprivation has been reported to increase both energy expenditure (e.g., through increased activity and stress responses) and food intake (Zheng et al., 2025). When increased energy expenditure temporarily exceeds compensatory intake, transient weight loss may occur. In the present study, the temporary reduction in body weight at week 8 followed by recovery may reflect such a transient imbalance.

It should be noted that the sleep deprivation protocol used in this study (rotating bar method) may induce not only sleep loss but also mild chronic stress. Stress itself is known to affect bone metabolism and body weight, potentially confounding the interpretation of our findings. Although we did not directly measure stress-related biomarkers in this study, several factors suggest that the observed effects are likely primarily related to sleep deprivation. First, the mice had free access to food and water throughout the intervention and were returned to standard cages for 6 hours of opportunity sleep each day, which may have partially mitigated stress. Second, previous studies using automated deprivation systems have reported minimal stress responses. For example, Dispersyn et al (Dispersyn et al., 2017) subjected C57BL/6 mice to 24 h of total sleep deprivation using a rotating wheel method and found no significant differences in corticosterone levels, adrenal gland weights, or body weights between sleep-deprived and control mice. Although the experimental setups are not identical, these findings provide indirect support that automated mechanical sleep deprivation methods may not induce substantial stress. These findings suggest that automated rotating deprivation methods do not induce severe stress. Nevertheless, the potential contribution of stress cannot be entirely excluded. Future studies should incorporate objective stress markers (e.g., corticosterone levels, adrenal weight) to better distinguish the relative effects of sleep deprivation and stress.

This study has important implications. By integrating morphological, quantitative and functional analyses, it provides comprehensive experimental evidence for further investigating SD—particularly chronic sleep insufficiency—as a potentially modifiable risk factor for OP in middle-aged and older men. The findings suggest that maintaining regular sleep patterns may have potential value in preventing age-related bone loss.

This study has several limitations. Firstly, the sample size was relatively small, which may have affected statistical power, particularly for detecting trends in biomechanical parameters. Secondly, a potential methodological confounder should be considered, as differences in lighting patterns and housing conditions existed between the two groups. These differences may differentially influence circadian rhythms and physiological stress responses, thereby potentially affecting bone metabolism independently of sleep deprivation. Although both groups shared the same photoperiod length (approximately 12 h) and ad libitum access to food and water, the possible confounding effects of lighting patterns and housing conditions cannot be entirely excluded. Future studies should standardise both artificial lighting and housing conditions across groups to minimise such potential biases. Thirdly, although morphological observations provide intuitive insight, they are inherently semiquantitative; future studies could incorporate bone histomorphometry to precisely quantify trabecular fractures, connectivity density and other structural parameters. Fourthly, this study included only a single endpoint (3 months), which precludes the characterisation of the dynamic changes in bone microarchitecture and mechanical properties over time. Fifthly, serum bone turnover markers (e.g. PINP and CTX) and relevant endocrine hormone levels were not measured, limiting the ability to elucidate the specific metabolic and molecular mechanisms underlying the observed alterations in bone structure. Sixthly, animal development was assessed only by weekly body weight measurements. We did not control daily food intake or measure nose-to-anus length, body surface area, or body mass index (BMI). These parameters could provide a more comprehensive assessment of metabolic and growth status. Future studies should incorporate these measurements to better evaluate the systemic effects of chronic sleep deprivation. Seventhly, despite considering body weight during group allocation, a significant baseline difference remained between the two groups (*P* < 0.001), likely due to the small sample size. This imbalance may have influenced the interpretation of longitudinal body weight changes. However, the primary outcomes of this study were based on micro-CT-derived bone parameters, which are not directly dependent on initial body weight. Therefore, this limitation is unlikely to have materially affected the main conclusions regarding the effects of chronic sleep deprivation on bone microarchitecture. Future studies should adopt body weight-matched or stratified randomisation to minimise such imbalance.

## 5 Conclusion

This study demonstrates that 3 months of CSD induces significant and visually discernible deterioration of bone microarchitecture in middle-aged male C57BL/6 mice, accompanied with declines in multiple bone parameters. Overall femoral biomechanical properties remain largely preserved, suggesting the presence of compensatory mechanisms in the skeleton. The effects of SD on body weight exhibit a fluctuating pattern. Future studies could extend the intervention duration, include additional dynamic observation points, incorporate detailed morphometric analyses and integrate assessments of bone turnover biomarkers and molecular biology to comprehensively elucidate the pathophysiological mechanisms by which CSD affects bone metabolism, thereby providing strong evidence for the potential role of sleep management in the primary prevention of OP.

## Data Availability

The raw data supporting the conclusions of this article will be made available by the authors, without undue reservation.

## Glossary

CSD: Chronic sleep deprivation;
BMD: Bone mineral density;
BV: Bone volume;
Tb.Th: Trabecular thickness;
Tb.Sp: Trabecular separation;
BS: Bone surface;
Tb.N: Trabecular number;
BV/TV: Bone volume fraction;
BS/BV: Bone surface area over bone volume;
OP: Osteoporosis;
SD: Sleep deprivation.
